# Lithium containing layered high entropy oxide structures

**DOI:** 10.1038/s41598-020-75134-1

**Published:** 2020-10-28

**Authors:** Junbo Wang, Yanyan Cui, Qingsong Wang, Kai Wang, Xiaohui Huang, David Stenzel, Abhishek Sarkar, Raheleh Azmi, Thomas Bergfeldt, Subramshu S. Bhattacharya, Robert Kruk, Horst Hahn, Simon Schweidler, Torsten Brezesinski, Ben Breitung

**Affiliations:** 1 Institute of Nanotechnology, Karlsruhe Institute of Technology (KIT), Hermann-von-Helmholtz-Platz 1, 76344 Eggenstein-Leopoldshafen, Germany; 2 Department of Materials and Earth Sciences, Technische Universität Darmstadt, Alarich-Weiss-Str. 2, 64287 Darmstadt, Germany; 3 Joint Research Laboratory Nanomaterials – Technische Universität Darmstadt and Karlsruhe Institute of Technology (KIT), Otto-Berndt-Str. 3, 64206 Darmstadt, Germany; 4 Institute for Applied Materials, Karlsruhe Institute of Technology (KIT), Hermann-von-Helmholtz-Platz 1, 76344 Eggenstein-Leopoldshafen, Germany; 5 Karlsruhe Nano Micro Facility (KNMF), Karlsruhe Institute of Technology (KIT), Hermann-von-Helmholtz-Platz 1, 76344 Eggenstein-Leopoldshafen, Germany; 6 Department of Metallurgical and Materials Engineering, Nano Functional Materials Technology Centre (NFMTC), Indian Institute of Technology Madras, Chennai, 600036 India; 7 Helmholtz Institute Ulm for Electrochemical Energy Storage, Helmholtzstr. 11, 89081 Ulm, Germany

**Keywords:** Chemistry, Energy science and technology, Materials science, Nanoscience and technology, Physics

## Abstract

Layered Delafossite-type Li_*x*_(M_1_M_2_M_3_M_4_M_5_…M_*n*_)O_2_ materials, a new class of high-entropy oxides, were synthesized by nebulized spray pyrolysis and subsequent high-temperature annealing. Various metal species (M = Ni, Co, Mn, Al, Fe, Zn, Cr, Ti, Zr, Cu) could be incorporated into this structure type, and in most cases, single-phase oxides were obtained. Delafossite structures are well known and the related materials are used in different fields of application, especially in electrochemical energy storage (e.g., LiNi_*x*_Co_*y*_Mn_*z*_O_2_ [NCM]). The transfer of the high-entropy concept to this type of materials and the successful structural replication enabled the preparation of novel compounds with unprecedented properties. Here, we report on the characterization of a series of Delafossite-type high-entropy oxides by means of TEM, SEM, XPS, ICP-OES, Mössbauer spectroscopy, XRD including Rietveld refinement analysis, SAED and STEM mapping and discuss about the role of entropy stabilization. Our experimental data indicate the formation of uniform solid-solution structures with some Li/M mixing.

## Introduction

Recently, the utilization of the high-entropy concept for oxide materials has been enjoying rising popularity^1–8^. High-entropy materials can be seen as compounds comprising several different elements in a single-phase structure and according to the Gibbs–Helmholtz equation, the resulting (large) configurational entropy may have structure stabilizing effects^9–14^. Additionally, the materials can be tailored with regard to their elemental composition, which leads to property changes due to the interactions between the different elements. The configurational entropy is solely based on the number of different elements incorporated in a single-phase structure (e.g., different species on the cationic sublattice) and can be calculated using a Boltzmann-entropy-derived equation (Gibbs entropy, Eq. 1).

$$S_{{{\text{config}}}} = - R\left[ {\mathop \sum \limits_{i = 1}^{N} x_{i} \ln x_{{i\;{\text{cation } - \text{ site}}}} + \mathop \sum \limits_{j = 1}^{M} x_{j} \ln x_{{j\;{\text{anion } - \text{ site}}}} } \right]$$

Equation 1: Calculation of configurational entropy.

In this formula, *S*_config_ is the configurational entropy, *R* the ideal gas constant and *x*_*i*_ and *x*_*j*_ represent the molar fraction of cations or anions on the respective sublattice. Regarding configurational entropy, an empirical value around 1.5*R* has been found, above which entropy-related effects appear and supposedly affect the material properties to various extents^15^. Although the term “high-entropy material” is formally used for every compound having a configurational entropy of > 1.5*R*, “entropy stabilization” can only be observed in certain high-entropy materials. A strong indication of entropy stabilization is the reversible mixing and demixing of phases upon increasing and decreasing temperature, respectively. This behavior was first reported by Rost et al*.* and can be used to classify materials into “high-entropy” and “entropy-stabilized” compounds^9,16^.

In this work, we report on a new class of high-entropy materials, namely Li_*x*_MO_2_ (M = metals), comprising five different equimolar and homogenously distributed cations. Li_*x*_MO_2_ materials crystallizing in the *R* − 3* m* space group (Delafossite α-NaFeO_2_-type structure) are known especially for their capability to reversibly intercalate Li^+^ ions. This characteristic made them become the most prominent cathode materials for Li-ion battery applications, e.g., LiCoO_2_ (LCO) and LiNi_*x*_Co_*y*_Mn_*z*_O_2_ (NCM), to name a few^17–20^. However, other areas are also in the focus of both academia and industry. For example, Delafossite-type materials can be used as semiconductors in solar cells, as catalysts for the photoelectrochemical reduction of CO_2_ as well as for thermoelectric and lighting applications^21–26^.

The layered high-entropy oxides (referred to as L-HEOs) were prepared by nebulized spray pyrolysis (NSP) and subsequent high-temperature annealing. Li precursor was used in excess in the synthesis to compensate for the Li (or Li_2_O) loss during heating. Specifically, five different compounds were successfully produced, namely, Li(NiCoMnAlZn)_1_O_2_, Li(NiCoMnAlFe)_1_O_2_ and Li_0.8_Na_0.2_(NiCoMnAlFe)_1_O_2_, with the metal ions present in equimolar proportions, and Li(NiCoMnMgAlCrTiZrCu)_1_O_2_ and Li(NiCoMnMgAlCrTiVCu)_1_O_2_ as non-equimolar compounds. The materials were characterized in detail, especially regarding the formation of solid-solution phases. Note that non-uniform elemental distribution would decrease the configurational entropy and therefore negatively affect the entropy stabilization. Possible entropic effects and the application of Hume Rothery’s second rules for solid solutions are discussed near the end of the manuscript for better understanding of the high-entropy concept^27^.

## Results and discussion

**Figure 1:**
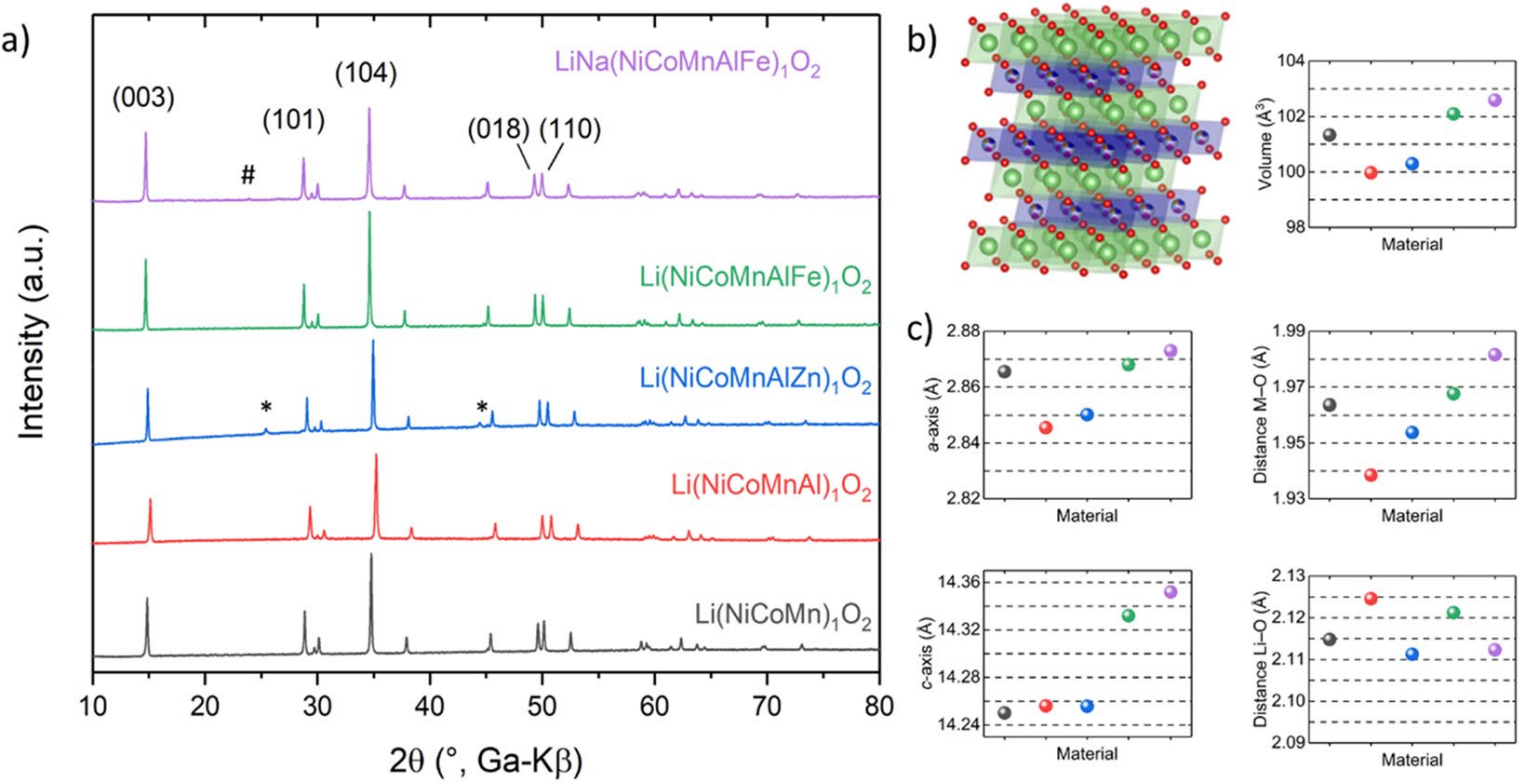
(**a**) XRD patterns for the different compounds, with secondary phases denoted by asterisks and number signs. (**b**) Crystal structure of α-NaFeO_2_ (drawing produced by Vesta 3, https://jp-minerals.org/). Green spheres represent lithium, red spheres oxygen and multi-colored spheres the positions of the statistically distributed metal cations. (**c**) Lattice parameters from Rietveld refinement analysis (color scheme as in panel a).

The L-HEO compounds were synthesized using a stepwise approach. In order to compare the structure and the structural evolution when incorporating more elements, two materials were prepared first using the same synthesis procedure. Then, additional elements were added in a stepwise manner and the resulting materials were characterized. All materials contain the metals in equimolar ratios. The study started from a typical NCM cathode material used for Li-ion battery applications, namely Li(NiCoMn)_1_O_2_ (see Fig. 1a). Then, Al was added, forming Li(NiCoMnAl)_1_O_2_, before entering into the high-entropy region with 5 or more different elements on the same cationic sublattice.

All samples showed a well-developed layered structure of α-NaFeO_2_ type (space group *R* − 3* m*, *Z* = 3, Fig. 1b), which can be seen from the clear splitting of the 018 and 110 reflections in the X-ray diffraction (XRD) patterns in Fig. 1a^28^. Alternating layering of metal and lithium layers occurred in all cases, with the metals and Li occupying the octahedral sites in the cubic close-packed oxygen lattice. The metals statistically occupy the Wyckoff 3*b* and Li the 3*a* sites. A possible intermixing was considered using Rietveld refinement analysis. The refined patterns are shown in the Supporting Information (Figure S1). Upon addition of Al to Li(NiCoMn)_1_O_2_, the reflections shifted to larger 2θ values, thus indicating a smaller unit cell. This result coincides with the smaller ionic radius of Al^3+^ (0.54 Å) than low-spin Ni^2+^ (0.69 Å), Ni^3+^ (0.56 Å) and Co^3+^ (0.55 Å) (the ionic radius of Mn^4+^ is 0.53 Å)^29^. This is also evident from the decreasing *a*-axis parameter (Fig. 1c), from 2.865(1) Å for Li(NiCoMn)_1_O_2_ to 2.845(1) Å for Li(NiCoMnAl)O_2_. Note that the *a*-axis is strongly affected by the ionic radius of the respective metals^30^. Because *V* = *a*^2^ · *c* · (sin 60°), there is also a strong decrease in unit-cell volume, from 101.333(9) Å^3^ for Li(NiCoMn)_1_O_2_ to 99.97(9) Å^3^ for Li(NiCoMnAl)_1_O_2_.

By adding Zn, an increase in *a*-axis parameter and unit-cell volume as well as formation of a secondary phase were observed (denoted by an asterisk in Fig. 1a; reflections indicated with number signs could not be assigned to a specific phase). The latter phase can most probably be assigned to a spinel-type structure, similar to ZnAl_2_O_4_. Note that spinels are known to form high-entropy structures when suitable ions are incorporated^31,32^. Therefore, a large number of possible compositions is conceivable (i.e., Zn(M_1_M_2_M_3_M_4_M_5_)_2_O_4_, with M = Zn, Fe, Co, Ni and/or Mn). In addition, Zn could be replaced by one or more (different) metal ions. Rietveld refinement assuming the presence of spinel revealed a ZnAl_2_O_4_ content of 9–10%. This value has to be taken with care, though, since we doubt that the reflection stems from “pure” ZnAl_2_O_4_. To further support our hypothesis, HR-TEM and SAED measurements were performed, which also indicated the presence of a distorted ZnAl_2_O_4_-type spinel structure (Figure S2).

Moreover, the addition of Zn led to an increase in the transition metal to oxygen distance, thereby corroborating its incorporation into the lattice. However, the position of the 104 reflection changed only slightly compared to that of Li(NiCoMnAl)_1_O_2_. Hence, it seems that Zn was only partially incorporated. This result emphasizes the importance of choosing suitable elements to maintain a single-phase structure.

Substituting Fe for Zn in Li(NiCoMnAlZn)_1_O_2_ led to an increase in lattice parameters, which again indicates successful incorporation into the crystal structure. In addition, the material was single phase, with the reflections shifted toward smaller angles. It is worth mentioning that the *a*-axis lattice parameter value for Li(NiCoMnAlFe)_1_O_2_ is similar to that of Li(NiCoMn)_1_O_2_. Based on the ionic radii, the unit cell was expected to increase.

**Figure 2:**
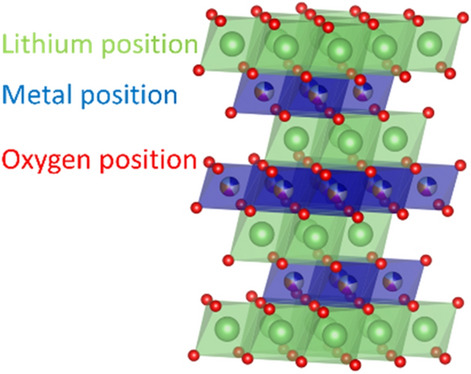
Lithium, metal and oxygen position in the Delafossite structure, as explained in Table 1 (drawing produced by Vesta 3, https://jp-minerals.org/).

The partial replacement of Li^+^ with Na^+^ (here, LiNa(NiCoMnAlFe)_1_O_2_), which has a considerably larger ionic radius (0.76 Å vs 1.02 Å), had a direct effect on the lattice parameters (increase in *a*, *c* and *V*, see Fig. 1c). For all L-HEOs, some Li/M cation mixing was apparent (see Table 1 and Fig. 2). Especially the Al-containing materials showed a relatively higher degree of mixing, with the exception of LiNa(NiCoMnAlFe)_1_O_2_ (most likely due to the presence of Na^+^).

**Table 1:** Li/M mixing from Rietveld refinement analysis (details in the Supporting Information).

	Lithium site			Metal site	
	Li	Na	Metal	Li	Metal
Li(NiCoMn)O_2_	0.96	0	0.04	0.15	0.85
Li(NiCoMnAl)O_2_	0.968	0	0.032	0.27	0.73
Li(NiCoMnAlZn)O_2_	0.965	0	0.035	0.26	0.74
Li(NiCoMnAlFe)O_2_	0.966	0	0.034	0.25	0.75
Li_0.8_Na_0.2_(NiCoMnAlFe)O_2_	0.8	0.2	0	0.11	0.89

The values are given in fractions of 1. Both Li(NiCoMn)_1_O_2_ and Li_0.8_Na_0.2_(NiCoMnAlFe)_1_O_2_ showed the lowest degree of cation mixing. The different positions are indicated in the crystal structure in Fig. 2.

**Figure 3:**
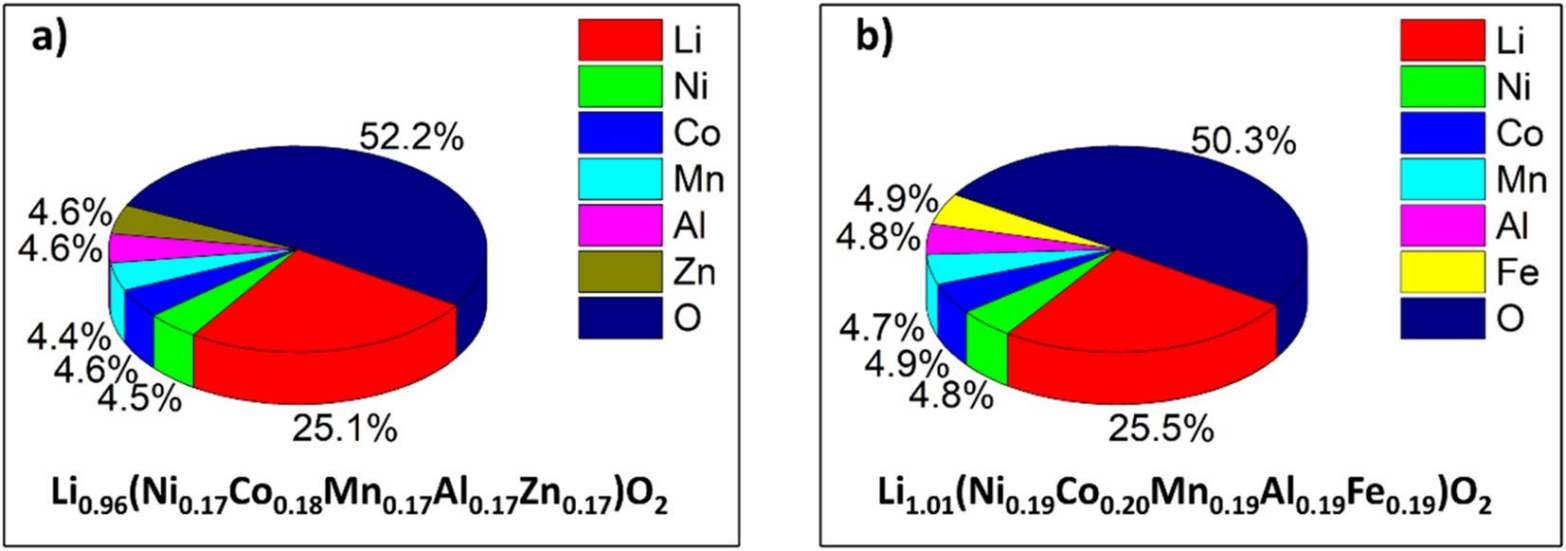
Chemical composition of (**a**) Li(NiCoMnAlZn)_1_O_2_ and (**b**) Li(NiCoMnAlFe)_1_O_2_ from ICP-OES analysis. The values are given in at%.

The composition of the different materials was examined by inductively coupled plasma-optical emission spectroscopy (ICP-OES). The corresponding results are shown in Fig. 3 and Figure S3, supporting the assumption that the metal ions are present in mostly equimolar proportions. Note that the data were normalized to an oxygen content of 2.

**Figure 4:**
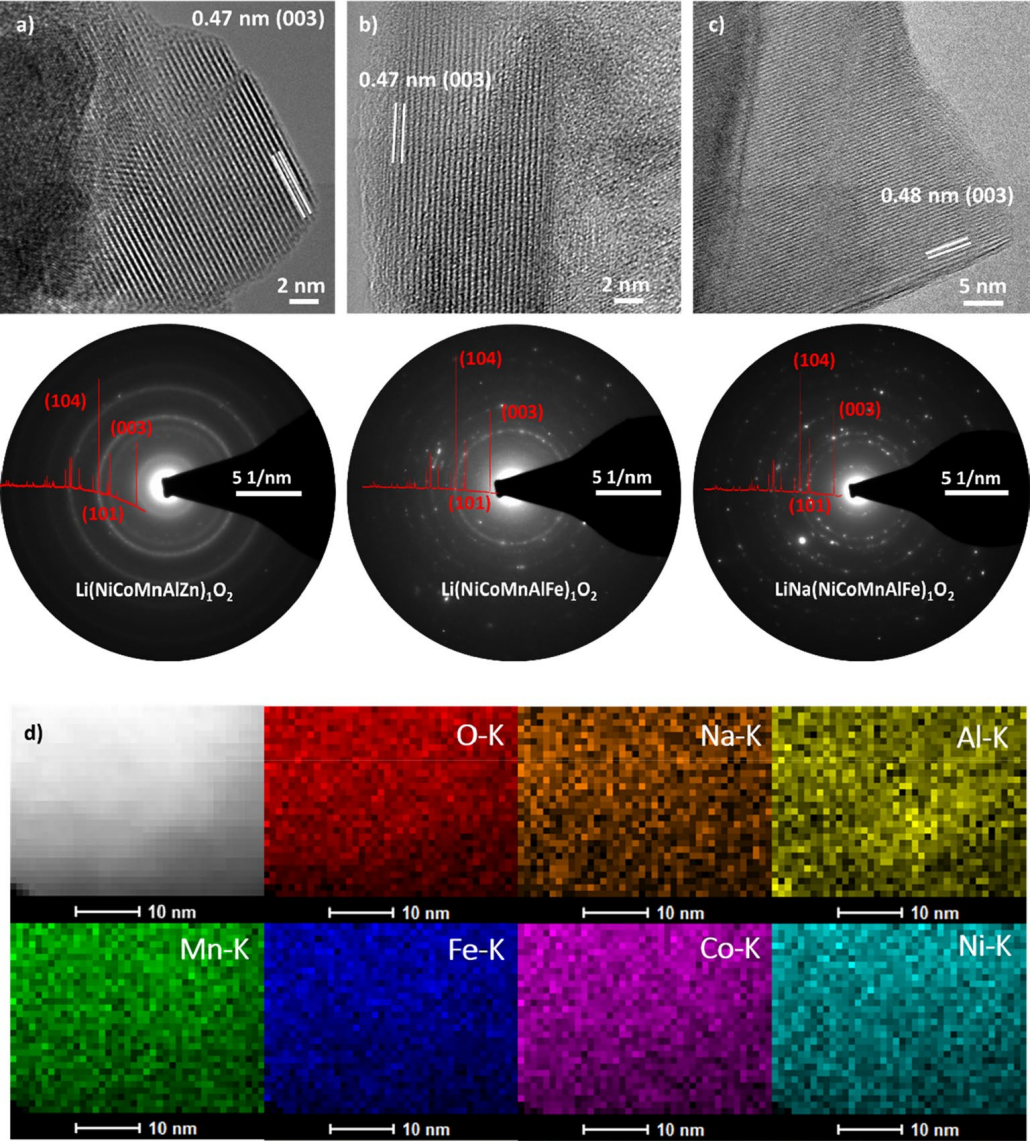
TEM of (**a**) Li(NiCoMnAlZn)_1_O_2_, (**b**) Li(NiCoMnAlFe)_1_O_2_ and (**c**) LiNa(NiCoMnAlFe)_1_O_2_. The HR-TEM micrographs emphasize the high degree of crystallinity and the SAED patterns corroborate the XRD results. (**d**) High-resolution EDX mapping of LiNa(NiCoMnAlFe)_1_O_2_, indicating uniform elemental distribution on the respective scale.

Scanning electron microscopy (SEM) and transmission electron microscopy (TEM) measurements were performed to gain more insights into the structure and the elemental distribution. Figure S4 shows SEM micrographs revealing particle sizes around 1 µm or below. These particles were agglomerated into larger aggregates. Figure 4a-c shows high-resolution TEM (HR-TEM) micrographs and selected-area electron diffraction (SAED) patterns for the different L-HEOs. The particles appear to be highly crystalline, irrespective of the composition and, for Li(NiCoMnAlZn)_1_O_2_, where a secondary phase was observed by XRD, a single-phase particle was probed. The Debye–Scherrer rings match the XRD data shown in Fig. 1a. The (003) lattice planes were also clearly detected by HR-TEM. Overall, the electron microscopy results confirm the layered structure of L-HEOs as well as the incorporation of elements into the Li_*x*_MO_2_ lattice.

Figure 4d presents results from energy dispersive X-ray spectroscopy (EDX) analysis of LiNa(NiCoMnAlFe)_1_O_2_. The mapping indicates uniform distribution of all elements on the nanometer level. No agglomeration or segregation of single elements or secondary phases were observed. This means that the largest possible configurational entropy can be expected. Assuming even distribution of elements and equal occupation probability over all sites in the structure, the configurational entropy was calculated to be 1.75*R* (1.5*R* for the L-HEOs without Na, see entropy calculation in the Supporting Information). Table S1 lists the calculated configurational entropy of all compounds.

To probe the distribution of elements over a larger sample area and to exclude clustering of elements, SEM/EDX measurements were performed. Figure S5 shows elemental heat maps obtained on a µm-size agglomerate of LiNa(NiCoMnAlFe)_1_O_2_ particles, corroborating the TEM results.

**Figure 5:**
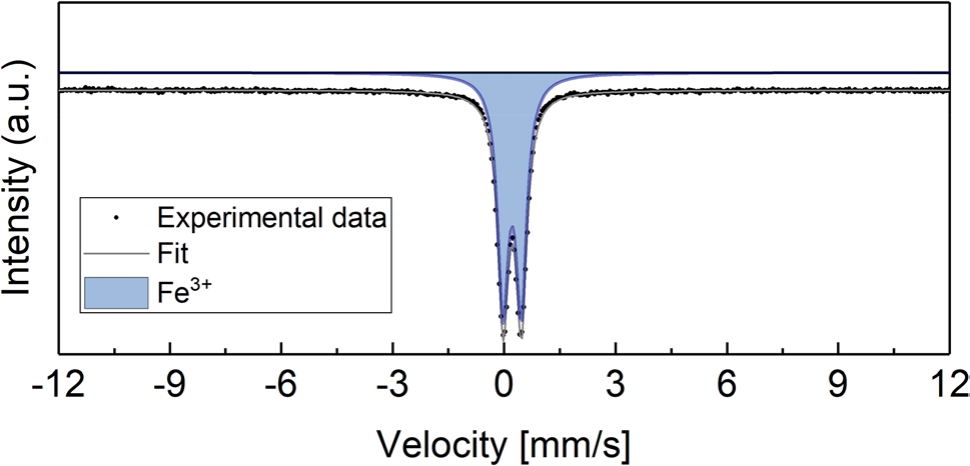
Mössbauer spectroscopy of LiNa(NiCoMnAlFe)_1_O_2_.

LiNa(NiCoMnAlFe)_1_O_2_ was further used to study the chemical environment and oxidation state of Fe by Mössbauer spectroscopy. This particular material was chosen because it contains the largest number of different elements, including Fe needed for Mössbauer spectroscopy. The spectrum shown in Fig. 5 was fitted by a quadrupole doublet with an average quadrupole splitting of 0.492 mm/s. The isomer shift (0.34 mm/s) suggests the presence of Fe^3+^. No other components were found, which would be indicative of different oxidation states and/or magnetic interactions and therefore inhomogeneities. Overall, the Mössbauer data demonstrate that the Fe^3+^ ions have a uniform chemical environment in the entire sample (approx. 150 mg) and, combined with the EDX results, they provide evidence of homogenous ion distribution and solid-solution state in the Li_*x*_MO_2_ lattice.

**Figure 6:**
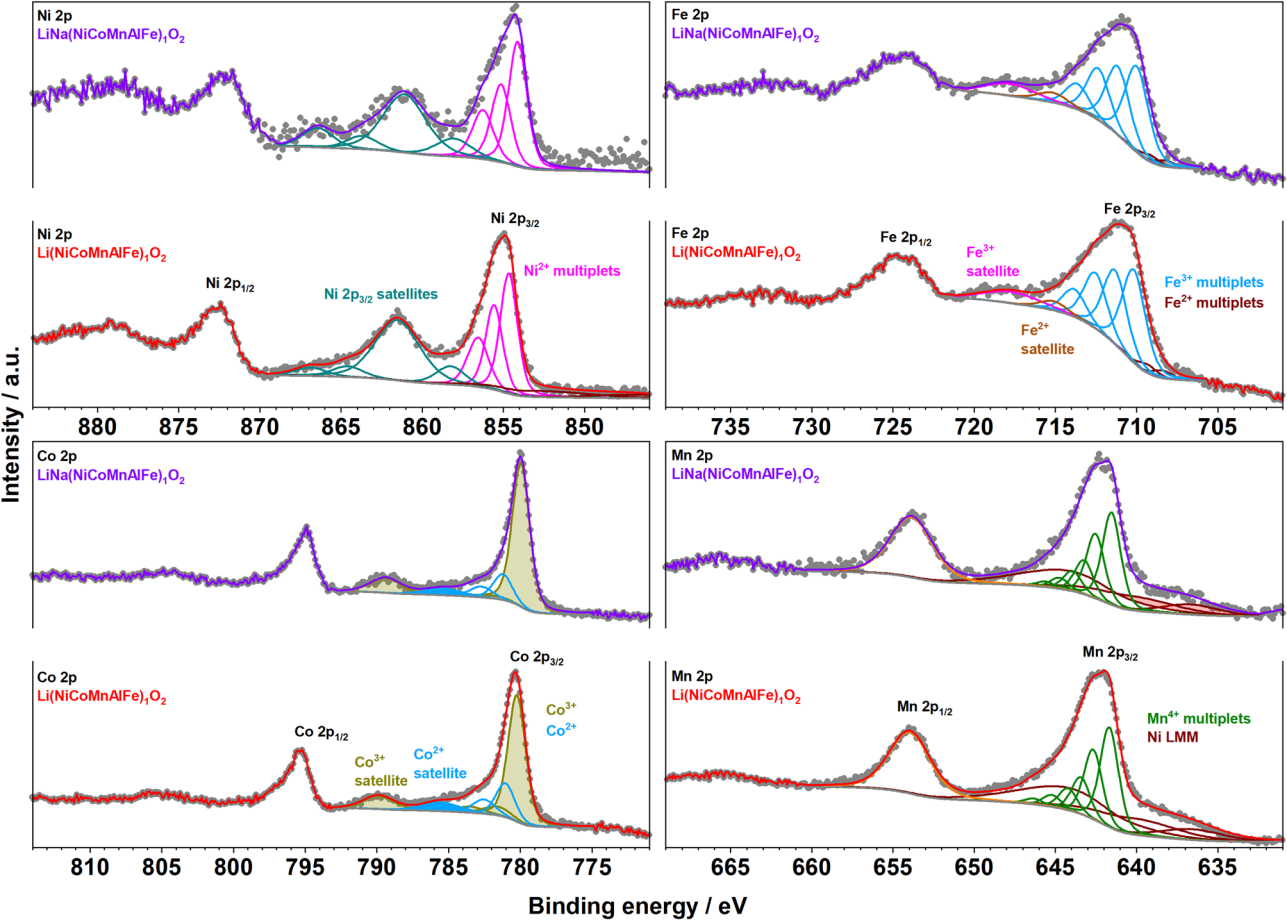
XPS of Li(NiCoMnAlFe)_1_O_2_ and LiNa(NiCoMnAlFe)_1_O_2_. The Ni, Co, Mn and Fe detailed spectra are shown.

X-ray photoelectron spectroscopy (XPS) was used to determine the oxidation state(s) of metals on the surface of the L-HEOs. In Fig. 6, the multiplet deconvolution of the main Ni 2p_3/2_ peaks of Li(NiCoMnAlFe)_1_O_2_ and LiNa(NiCoFeMnAl)_1_O_2_ are shown in magenta and their pronounced Ni 2p satellite in cyan. According to the presented deconvolution (based on our previous publications on NCM materials), Ni ions were identified as Ni^2+^^33,34^. The Co ions in both Li(NiCoFeMnAl)_1_O_2_ and LiNa(NiCoMnAlFe)_1_O_2_ showed a minor contribution of Co^2+^, according to the low intensity peak detected around 785.5 eV that is considered a characteristic satellite of Co^2+^ ions (shown in blue in Fig. 6)^33,34^. The majority of Co ions were identified as Co^3+^ (shown in dark yellow). The ratio of Co^2+^/Co_total_ was determined as 30% and 24% for Li(NiCoMnAlFe)_1_O_2_ and LiNa(NiCoMnAlFe)_1_O_2_, respectively.

The Mn 2p_3/2_ spectra could be deconvoluted with a Mn^4+^ multiplet structure (green color) and including the Ni LMM Auger contribution (in dark brown color)^33,34^. This deconvolution is the base of assignment of the 4+ state to Mn ions in L-HEOs. The use of Mn 3p and Mn 3s spectra for identification of the Mn oxidation state was hampered due to overlap of these peaks with Fe 3p and Li 1s and Fe 3s (see also Figure S6). The overlapping Ni LMM and Co LMM Auger peaks increased the background of the Fe 2p spectra and added to the difficulty of deconvoluting the spectra for oxidation state identification. However, according to the presented multiplet splitting (deconvolution of the Fe 2p_3/2_ spectra and the specified satellite structure, shown in blue), the Fe ions were identified mainly as Fe^3+^ and the contribution of the Fe^2+^ state was negligible (less than 8% of total Fe ions, explainable for surfaces under air and fitting difficulties because of overlapping peaks)^35,36^. The Al 2p spectra of L-HEOs overlapped with the Ni 3p spectra (the Al 2s also overlapped with Ni 3s). Our attempt to properly deconvolute the Al 2p spectra resulted in the peak fitting shown in Figure S6. The binding energy of Al 2p_3/2_ was 72.8 and 74 eV for Li(NiCoMnAlFe)_1_O_2_ and Li_0.8_Na_0.2_(NiCoMnAlFe)_1_O_2_, respectively. The differences in the binding energy of Al can be explained by the diverging cation mixing of the respective materials. Additionally, the very oxophilic nature of Al might result in the formation of oxide species on the surface, which are a bit different, depending on the composition of the layered structure^37^.

**Figure 7:**
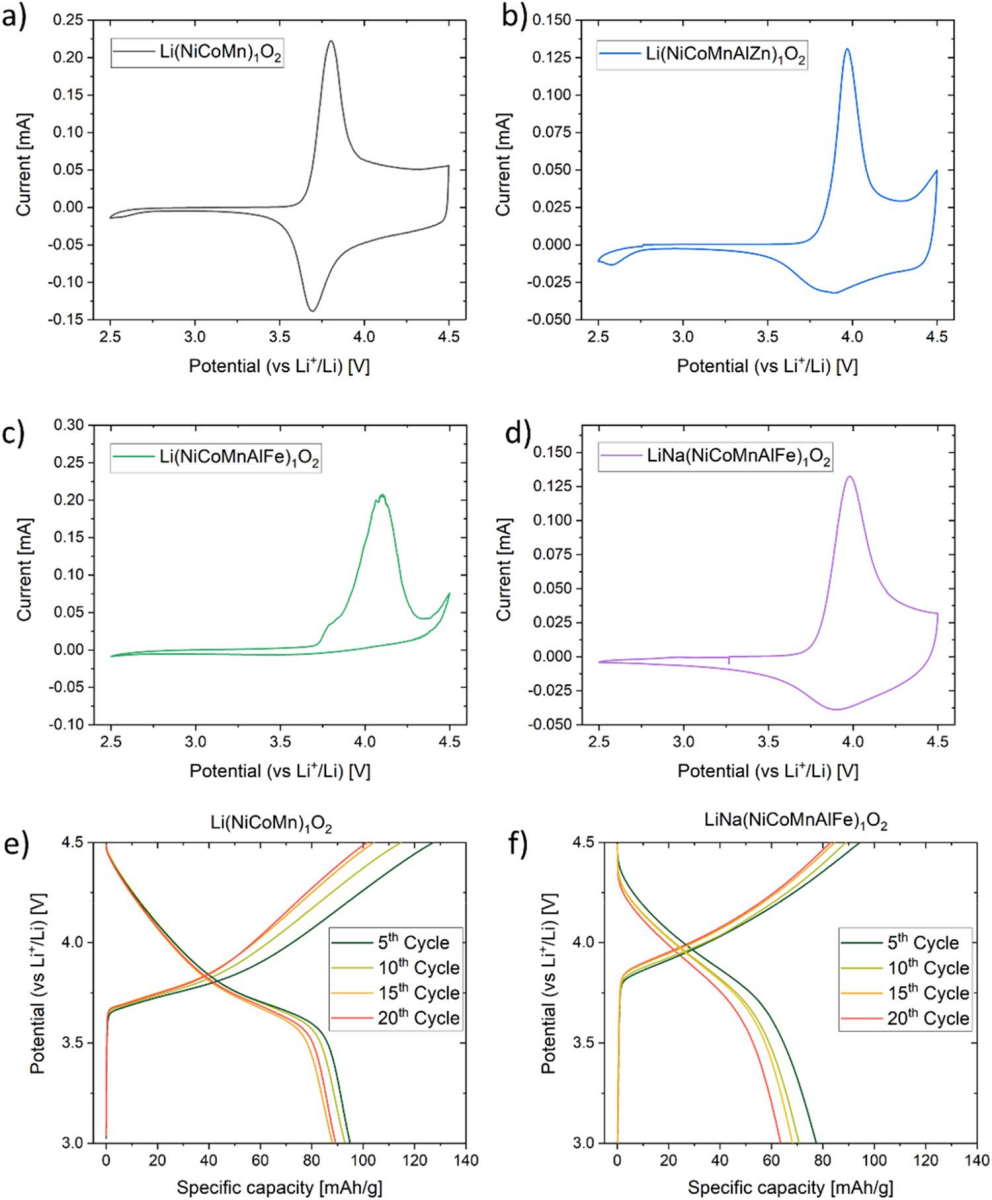
Cyclic voltammetry and charge/discharge profiles of as prepared (**a**) Li(NiCoMn)_1_O_2_, (**b**) Li(NiCoMnAlZn)_1_O_2_, (**c**) Li(NiCoMnAlFe)_1_O_2_ and (**d**) LiNa(NiCoMnAlFe)_1_O_2_. The charge and discharge profiles for Li(NiCoMn)_1_O_2_ and LiNa(NiCoMnAlFe)_1_O_2_ are shown in (**e**) and (**f**), respectively.

Recently, Zhao et al.^38^ showed for Na-ion batteries that the high-entropy concept is beneficial for achieving stable cell capacities. Because NCM-type materials are common cathodes for Li-ion batteries, in the present work, the L-HEO structures were tested regarding the possibility to reversibly intercalate Li^+^ ions (Fig. 7). For comparison, Li(NiCoMn)_1_O_2_ prepared under the same conditions served as a kind of reference cathode material. Most of the materials showed reasonable redox activity, but the specific capacities achieved are not yet satisfactory (Figure S7), probably because of Li/M cation mixing (note that the morphology has not yet been optimized), blocking pathways for Li transport. Figure 7a-d shows cyclic voltammetric curves at a sweep rate of 0.1 mV/s. The Li(NiCoMn)_1_O_2_ data revealed an oxidation peak around 3.8 V, in agreement with literature. The potential increased to 4.0 V for Li(NiCoMnAlZn)_1_O_2_. However, when substituting Fe^3+^ for Zn^2+^ [Li(NiCoMnAlFe)_1_O_2_], it increased even further to 4.1 V, despite the fact that the Al^3+^ content remained unchanged. Although this material did not show high reversibility, the increase in oxidation potential clearly demonstrates that either the incorporated elements or the configurational entropy or both exert a strong effect on the electrochemical behavior. Interestingly, when some of the Li was replaced with Na [LiNa(NiCoMnAlFe)_1_O_2_], both the specific capacity increased by a factor of more than two (Figure S7) and the reversibility improved considerably compared to Li(NiCoMnAlFe)_1_O_2_. The oxidation potential dropped by only 100 mV to 4.0 V, which seems promising for the development of high-energy-density cathode materials. Figure 7e,f shows the voltage profiles of cells using Li(NiCoMn)_1_O_2_ and LiNa(NiCoMnAlFe)O_2_ over the first 20 cycles, corroborating the cyclic voltammetry results.

**Figure 8:**
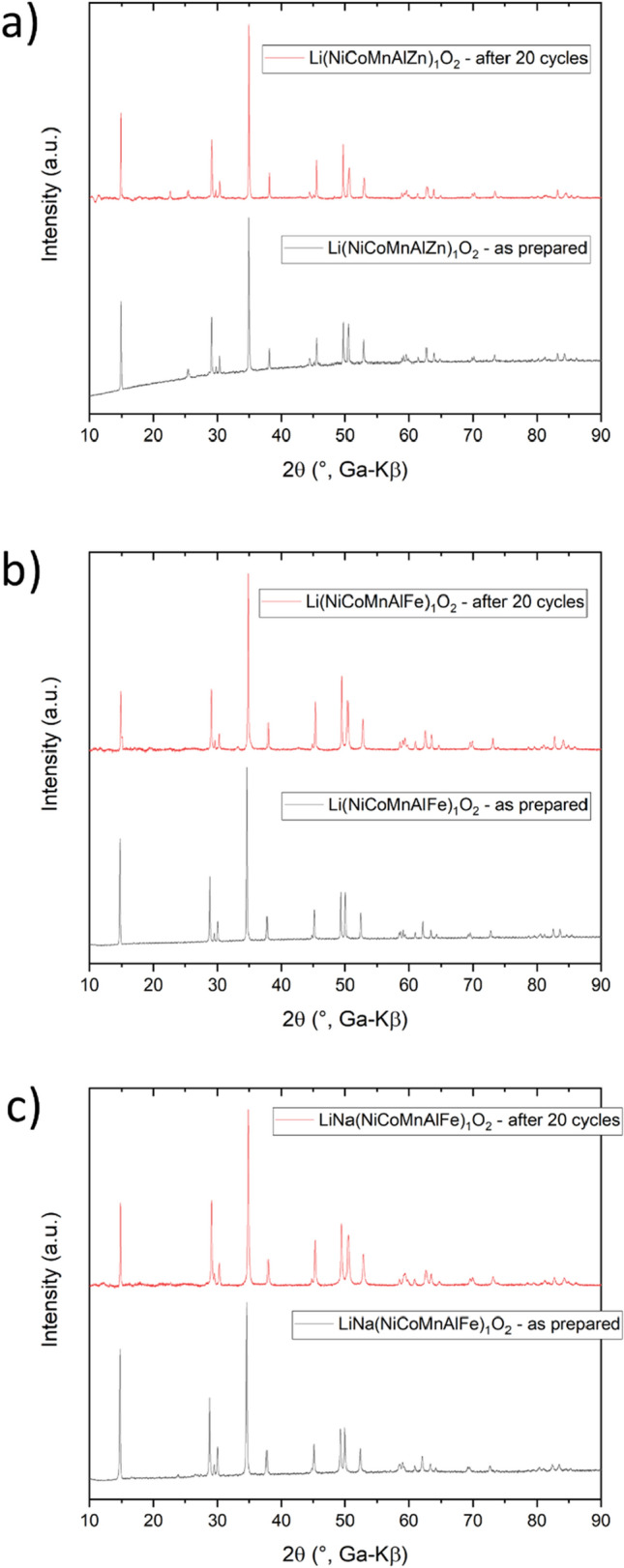
XRD patterns of (**a**) Li(NiCoMnAlZn)_1_O_2_, (**b**) Li(NiCoMnAlFe)_1_O_2_ and (**c**) LiNa(NiCoMnAlFe)_1_O_2_ recorded before and after electrochemical cycling.

In order to learn about the structural stability upon electrochemical cycling, the L-HEO materials were probed using XRD before and after 20 cycles (Fig. 8a–c). The layered structure was clearly preserved for all of the materials, thus indicating good stability with cycling. Only for Li(NiCoMnAlZn)_1_O_2_, a small reflection appeared around 23°. What is more striking is the change in intensity ratio between the 003 and 104 reflections. If the reflections do show about the same intensity, the cation mixing is low. For cycled Li(NiCoMnAlFe)_1_O_2_ and LiNa(NiCoMnAlFe)_1_O_2_, the 003 reflection showed decreased intensity, suggesting increased cation mixing, which in turn may help explain the capacity fading shown in Figs. 7 and S7.

Taken together, the data in Figs. 7 and 8 demonstrate the versatility of the high-entropy approach—small changes in composition strongly affect the electrochemical characteristics, including cycling performance and stability. The reason for the enhanced performance of LiNa(NiCoMnAlFe)_1_O_2_ is unclear at this time, but it may well be that the larger Na^+^ ions somewhat widen the diffusion channels that are blocked otherwise due to cation disorder^39^. As somewhat expected, the L-HEO material with the lowest degree of cation mixing showed the best cyclability and delivered the largest specific capacities. This suggests that, in principle, it should be possible to design next-generation L-HEOs for battery applications^38,40,41^. Nevertheless, the issue of cation mixing remains to be resolved and the specific capacities need to be increased^42–44^. Overall, the impact of entropy stabilization on the cycling performance is largely unclear and is an interesting question for future studies. Common (non-HEO) cathode intercalation materials can only be delithiated to a certain extent to prevent irreversible structural changes^45,46^. This reduces the amount of usable Li and therefore limits the specific capacity. Overall, it would be exciting to see if L-HEO materials with a large entropy stabilization allow for higher degrees of delithiation by more effectively stabilizing the layered structure.

**Figure 9:**
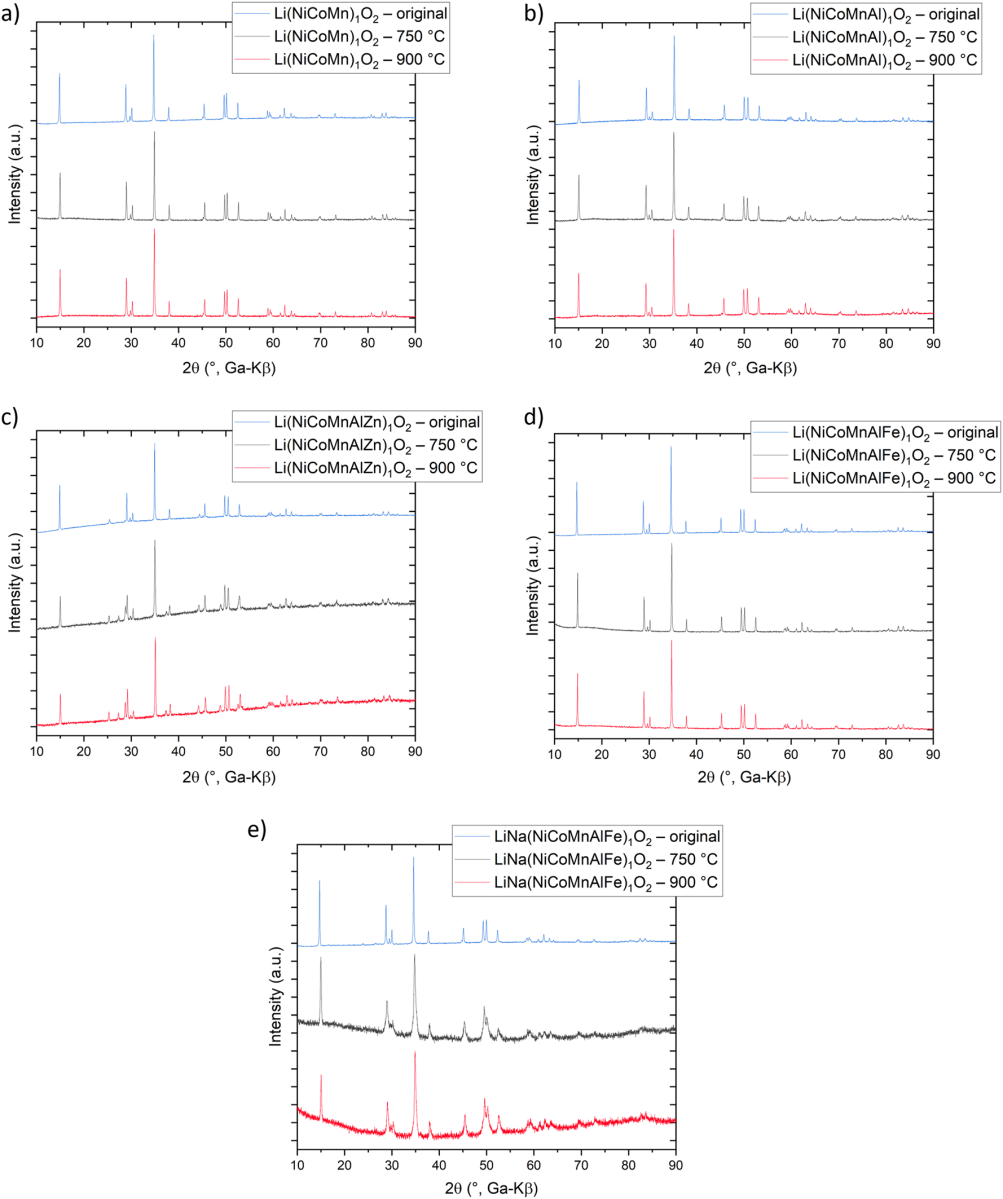
Heating tests of (**a**) Li(NiCoMn)_1_O_2_, (**b**) Li(NiCoMnAl)_1_O_2_, (**c**) Li(NiCoMnAlZn)_1_O_2_, (**d**) Li(NiCoMnAlFe)_1_O_2_ and (**e**) LiNa(NiCoMnAlFe)_1_O_2_.

Making statements about possible entropy stabilization in the layered structures is difficult. Experiments to elucidate any potential entropy stabilization, like the reversible phase separation at low cooling rates, as shown by Rost et al*.* and others, are impeded by the Li (or Li_2_O) evaporation at high temperatures. This loss can lead to phase changes, as known for NCM cathode materials (the layered structure transforms into spinel and/or rock salt at high states of charge/low lithium content). Moreover, some materials are only stable because the Li^+^ ions compensate higher valence charges, which would break the charge neutrality of the structure, thus leading to phase separation. Nevertheless, experiments to determine the reaction(s) of the compounds when heated to a lower temperature and cooled down slowly were performed. Specifically, the L-HEO materials were heated to 750 °C for 3 h to trigger (phase) demixing and then cooled down slowly. Afterwards, the materials were heated to 900 °C for 3 h to allow the phases to mix again. Figure 9a shows the results for Li(NiCoMn)_1_O_2_, where no differences in the XRD patterns were observed. Li(NiCoMnAl)_1_O_2_ also did not show any effect of temperature and/or Li loss on the structure (Fig. 9b). These materials exhibit a configurational entropy below the 1.5*R* threshold and therefore do not fall under the description of high-entropy materials. The configurational entropy is too small to compensate for any mixing enthalpy penalties, so the structure itself seems to be stable (without entropy stabilization). Hence, it is not surprising that the temperature does not affect the structure. Figure 9c shows the corresponding XRD patterns for Li(NiCoMnAlZn)_1_O_2_. Upon heating to 750 °C, additional reflections appeared, which can be assigned to a distorted ZnO (Wurtzite) phase. The latter reflections did not disappear but rather grew in intensity when heating the material to 900 °C. We attribute this to demixing at 750 °C and the Li loss at 900 °C, leading to phase separation of the original material. As expected, the reflections of Li(NiCoMnAlZn)_1_O_2_ also shifted slightly toward smaller lattice parameters. Moreover, the intensity ratio between the 003 and 104 reflections changed, indicating stronger cation mixing. For Li(NiCoMnAlFe)_1_O_2_, despite being a high-entropy material, again no differences in the XRD patterns were observed (Fig. 9d). Perhaps, this is due to a significantly lower enthalpy of mixing. When Zn^2+^ is replaced with Fe^3+^, the average charge is changing toward the ideal one in the α-NaFeO_2_ type structure. In addition, the ionic radius of Fe^3+^ is closer to that of Co^3+^ and Ni^3+^, all of which probably reduces the enthalpy of mixing, so that entropy stabilization is not “required” anymore (similar to both Li(NiCoMn)_1_O_2_ and Li(NiCoMnAl)_1_O_2_). For LiNa(NiCoMnAlFe)O_2_, the reflections decreased in intensity and broadened significantly (Fig. 9e), indicative of major structural distortions. We attribute this to the introduction of Na and its properties. In this case, the enthalpy of mixing is clearly increased, due to the different ionic radii and electronegativities.

The question if “only” a high-entropy or an entropy stabilization is apparent can be discussed in part using the Hume-Rothery’s second rule for solid-solution formation^45^. The latter can be understood as a description under which precondition elements can form a solid solution. The rule states that the ionic radius of the incorporated elements should not differ by more than about 15% and that the crystal structures, valences and electronegativities should be similar. The type of crystal structure of the L-HEO materials synthesized here is Delafossite α-NaFeO_2_. Many of the individual elements may show comparable stable structures in the respective lattice type (α-LiAlO_2_, LiCoO_2_, LiNiO_2_, LiFeO_2_, except for Mn and Zn). Therefore, the first precondition seems to be fulfilled for 4 out of 6 of the tested elements. Other preconditions are similar ionic radii and electronegativities. Table 2 shows the respective values. As is evident, these characteristics are similar for the trivalent transition metal ions. It is known that in NCM materials, Mn is present in the oxidation state 4+ , thereby partially reducing Ni to 2+ . The size of Mn^4+^ is also similar, which helps explain its successful incorporation into a single-phase structure. The radius of Zn^2+^ differs quite significantly and is more similar in radius to Li^+^. The discrepancy in Zn^2+^ radius and the differences in structure as explained above seem to increase the mixing enthalpy too much, such that the entropy cannot compensate it and therefore no single-phase material is formed. Nevertheless, XRD and TEM indicate that some of the Zn^2+^ ions are incorporated into the material, so entropy stabilization and the formation of a single-phase structure may be apparent until a certain threshold concentration in the structure is reached. Formation of a single phase at higher temperatures may be possible, but the accompanying Li loss prevents such studies. Despite its even larger radius and discrepancy in valence and electronegativity, Li^+^ is successfully incorporated, since it is an integral part of the layered *R* − 3* m* structure.

**Table 2:** Valence, ionic radius and electronegativity for different elements^47,48^.

	Co^3+^	Ni^2+^	Ni^3+^	Al^3+^	Fe^3+^	Mn^4+^	Zn^2+^	Li^+^	Na^+^
Ionic radius (Å)	0.55	0.69	0.56	0.54	0.55	0.53	0.74	0.76	1.02
Electronegativity	1.693	1.367	1.695	1.513	1.651	1.912	1.336	1.009	1.024

In summary, application of the Hume-Rothery rules to these systems indicates good miscibility of elements for Li(NiCoMnAlFe)_1_O_2_ and LiNa(NiCoMnAlFe)_1_O_2_. In these systems, high-entropy is clearly apparent, but entropy stabilization might be questionable. The Li(NiCoMnAlZn)_1_O_2_ material showed the presence of side phases, most probably due to the different characteristics of Zn^2+^. Therefore, the stabilizing role of entropy remains elusive for these layered materials and the term “high-entropy” (referring to high configurational entropy) rather than “entropy stabilized” has been applied in the present work^16^.

**Figure 10:**
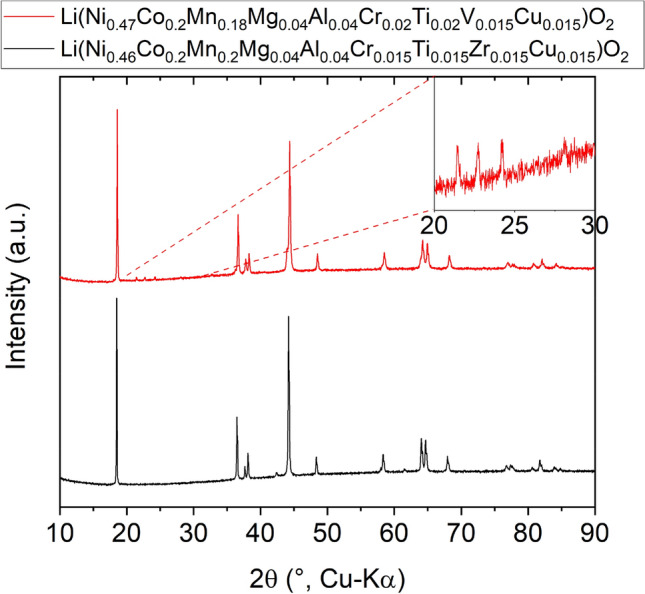
XRD patterns of Li(Ni_0.47_Co_0.2_Mn_0.18_Mg_0.04_Al_0.04_Cr_0.02_Ti_0.02_V_0.015_Cu_0.015_)O_2_ (in red) and Li(Ni_0.46_Co_0.2_Mn_0.2_Mg_0.04_Al_0.04_Cr_0.015_Ti_0.015_Zr_0.015_Cu_0.015_)O_2_ (in black). The inset shows the additional reflections arising from the slight change in cation stoichiometry.

Two more compositions are presented to emphasize the versatility of the high-entropy concept as well as to demonstrate how sensitive this system is toward small changes in stoichiometry. As can be seen in Fig. 10, the layered structure has clearly formed. However, upon slightly altering the Ni, Mn, Cr and Ti concentrations, a secondary phase appeared (see minor reflections between 20 and 25° 2θ, most probably lithiated V_2_O_5_ compounds, like Li_0.3_V_2_O_5_ or Li_0.6_V_2_O_5_), probably because the average valence of cations changed to some extent. This is in accordance with the Hume-Rothery’s second rule, which states that the valences should be similar. Additionally, charge neutrality has to be maintained, which forces the compound to form vacancies or to undergo structural changes. The same behavior can be assumed when Zn^2+^ is incorporated instead of Fe^3+^, as shown before. This may be a possible explanation for the multiphase nature of Li(NiCoMnAlZn)_1_O_2_. The results from heating experiments are shown in Figure S8, indicating strong cation mixing upon subsequent heating.

In summary, a new class of high-entropy oxide materials, namely layered Li_*x*_MO_2_, has been synthesized. The incorporated elements were found to be virtually equimolar and, more importantly, uniformly distributed on the nanometer and micrometer levels. The calculated configurational entropy amounted up to 1.75*R*, rendering these compounds high-entropy materials. The question if these materials are entropy-stabilized cannot be clearly answered due to Li evaporation at elevated temperatures. Moreover, reversible Li^+^ intercalation/extraction into/from the L-HEOs was demonstrated, but the specific capacities achieved were relatively low (when compared to state-of-the-art cathode materials). Nevertheless, given the broad application area of layered materials and the benefits arising from the high-entropy approach (e.g., versatility in composition), L-HEOs may pave the way for new technologies.

## Methods

### Synthesis

The compounds were synthesized by NSP (see Figure S9 and details in the Supporting Information). First, nitrate salts of the respective metals (LiNO_3_, Co(NO_3_)_2_·6H_2_O, Ni(NO_3_)_2_·6H_2_O, Mn(NO_3_)_2_·4H_2_O, Zn(NO_3_)_2_·6H_2_O, Fe(NO)_3_·9H_2_O, Al(NO_3_)_3_·6H_2_O, NaNO_3_; from Sigma-Aldrich) were dissolved in 200 mL of H_2_O to form a 0.1 M solution. 10 wt% excess LiNO_3_ was used to compensate for the Li (or Li_2_O) evaporation during heating. The resultant clear solution was sprayed as a mist and transported by means of a carrier gas into the hot zone of a tubular furnace (800 °C). The as-made powder was quenched and collected. After the NSP synthesis process, all collected powder was further calcined at 900 °C for 12 h using a heating rate of 5 °C/min and then cooled down. Li(Ni_0.47_Co_0.2_Mn_0.18_Mg_0.04_Al_0.04_Cr_0.02_Ti_0.02_V_0.015_Cu_0.015_)O_2_ and Li(Ni_0.46_Co_0.2_Mn_0.2_Mg_0.04_Al_0.04_Cr_0.015_Ti_0.015_Zr_0.015_Cu_0.015_)O_2_ were prepared by solid-state synthesis. Stoichiometric amounts of LiNO_3_, NiO, Co_3_O_4_, MnO, MgO, Cr(NO_3_)_3_·9H_2_O, Al_2_O_3_, V_2_O_5_, CuO, TiO_2_ and ZrC_20_H_28_O_8_ were thoroughly mixed by ball milling (300 rpm) for 10 h with ethanol. After drying, the mixed precursors were annealed at 900 °C for 12 h.

### Electrode Processing

For the cathode preparation, 7:2:1 by weight of active material, Super C65 carbon black additive (Timcal) and PVDF binder (Solef 5130) were used. The different constituents were uniformly dispersed into *N*-Methyl-2-pyrrolidone (NMP) using a planetary mixer (Thinky). Next, the slurry was cast onto an Al current collector and dried in a vacuum at 80 °C for 12 h. Circular electrodes of 13 mm diameter were cut from the cathode sheet, assembled inside an Ar-filled glovebox and tested using CR2032 coin cells. 1 M LiPF_6_ in 3:7 by weight of ethylene carbonate (EC) and ethyl methyl carbonate (EMC; Selectilyte LP57, BASF SE), GF/C glass microfiber filter paper (Whatman) and Li metal foil (Gelon LIB Co., Ltd) were used as electrolyte, separator and counter electrode, respectively. The electrode active material loading was around 1 mg/cm^2^. The specific capacity was calculated on the basis of the active material mass.

### Instrumentation

TEM measurements were conducted on powder samples dispersed on a lacey carbon-coated copper grid. The samples were loaded onto an FEI double tilt holder. TEM, SAED and STEM-EDX data were collected using an FEI Titan 80-300 microscope equipped with a CEOS image spherical aberration corrector, a HAADF STEM detector (Fischione model 3000), EDAX SUTW EDX detector and a Tridiem Gatan image filter. The microscope was operated at an accelerating voltage of 300 kV.

XPS measurements were performed on a K-Alpha+ instrument (Thermo Fisher Scientific) with a monochromatic Al-Kα X-ray source (1486.6 eV) and 400 μm spot size. The K-Alpha + charge compensation system was applied to prevent localized charge buildup during analysis using 8 eV electrons and low-energy Ar ions. Data acquisition and processing were carried out using the Thermo Avantage software^49^. The spectra were fitted with one or more Voigt profiles. The binding energies are given with respect to that of the C 1 s peak of hydrocarbons at 285.0 eV. The analyzer transmission function, Scofield sensitivity factors and effective attenuation lengths (EALs) for photoelectrons were applied for quantification^50^. EALs were calculated using the standard TPP-2 M formalism^51^. The multiplet splitting effect of Ni ions and the difficulty of identifying their oxidation state solely based on the peak barycenter have already been reported^36,52,53^.

SEM was performed on a ZEISS Gemini Leo 1530, equipped with an Oxford EDX detector.

XRD measurements were conducted on powder samples using either a STOE Stadi P diffractometer equipped with a Ga-jet X-ray source (Ga-Kβ radiation) or a Bruker D8 Advance diffractometer with a Cu-Kα radiation source and a LYNXEYE detector of fixed divergence slit (0.3°). Refinement of XRD patterns was performed using TOPAS Academics V5 software. Strain-free Al_2_O_3_ in a glassy matrix (particle size > 2 µm, random orientation) served as calibration sample to determine the instrumental resolution. The latter calibration sample is commercially available and was received from NIST. A linear interpolation function with 36 parameters was used for background refinement.

For ICP-OES spectroscopy (iCAP 7600 ICP-OES DUO; Thermo Fisher Scientific), an amount of about 10 mg of the samples (weighing accuracy ± 0.05 mg) was dissolved in 6 ml of hydrochloric acid, 2 ml of nitric acid and 4 ml of sulfuric acid at 513 K for 35 min in a microwave oven (Speedwave Xpert from Berghof). The analysis of elements was accomplished with 4 different calibration solutions and an internal standard (Sc). Two or three major wavelengths of elements were used for the calculation. The oxygen content was determined by the missing weight and compared to the oxygen content analyzed using the method of carrier gas hot extraction (CGHE). To this end, a commercial oxygen/nitrogen analyzer TC600 (LECO) was used. The differences were in the accuracy range of the CGHE method. The accuracy of ICP is usually very high and on average around + /− 0.2–0.5% for the metals, depending on the mass fraction of the element. Therefore, we included all metals with a mass fraction below 0.5% in a combined segment called “other elements”.

^57^Fe Mössbauer spectroscopy was performed in transmission geometry using a spectrometer with a moving source of ^57^Co in an Rh matrix and a triangular velocity variation. The isomer shift is given relative to bcc-Fe at room temperature.

Galvanostatic charge/discharge measurements were performed using an Arbin battery test system (BT-2000) at room temperature.

## Supplementary information

Supplementary Information

## Data Availability

The datasets generated during and/or analyzed during the current study are available from the corresponding authors on reasonable request.
