# Supplementary material for: Lithium containing layered high entropy oxide structures
Source: Sci Rep. 2020 Oct 28;10:18430. doi: 10.1038/s41598-020-75134-1 (PMC7595184; doi:10.1038/s41598-020-75134-1)
Supplement: Supplementary file 1 — Supplementary Information [file 41598_2020_75134_MOESM1_ESM.docx]

**Supplementary Information (SI)**

**Lithium Containing Layered High Entropy Oxide Structures**

Junbo Wang,^a^ Yanyan Cui,^a^ Qingsong Wang,^a^ Kai Wang,^b^ Xiaohui Huang,^b^ David Stenzel,^a^ Abhishek Sarkar,^a,c^ Raheleh Azmi,^d^ Thomas Bergfeldt,^e^ Subramshu S. Bhattacharya,^f^ Robert Kruk,^a^ Horst Hahn,^a,c,g^ Simon Schweidler,^a^ Torsten Brezesinski ^a,*^ and Ben Breitung ^a,*^

^a^ Institute of Nanotechnology, Karlsruhe Institute of Technology (KIT), Hermann-von-Helmholtz-Platz 1, 76344 Eggenstein-Leopoldshafen, Germany

^b^ Department of Materials and Earth Sciences, Technische Universität Darmstadt, Alarich-Weiss-Str. 2, 64287 Darmstadt, Germany

^c^ Joint Research Laboratory Nanomaterials – Technische Universität Darmstadt and Karlsruhe Institute of Technology (KIT), Otto-Berndt-Str. 3, 64206 Darmstadt, Germany

^d^ Institute for Applied Materials, Karlsruhe Institute of Technology (KIT), Hermann-von-Helmholtz-Platz 1, 76344 Eggenstein-Leopoldshafen, Germany

^e^ Karlsruhe Nano Micro Facility (KNMF), Karlsruhe Institute of Technology (KIT), Hermann-von-Helmholtz-Platz 1, 76344, Eggenstein-Leopoldshafen, Germany

^f^ Department of Metallurgical and Materials Engineering, Nano Functional Materials Technology Centre (NFMTC), Indian Institute of Technology Madras, Chennai, 600036, India

^g^ Helmholtz Institute Ulm for Electrochemical Energy Storage, Helmholtzstr. 11, 89081 Ulm, Germany

* Corresponding author/s, Correspondence to [ben.breitung@kit.edu](mailto:ben.breitung@kit.edu), [torsten.brezesinski@kit.edu](mailto:torsten.brezesinski@kit.edu)


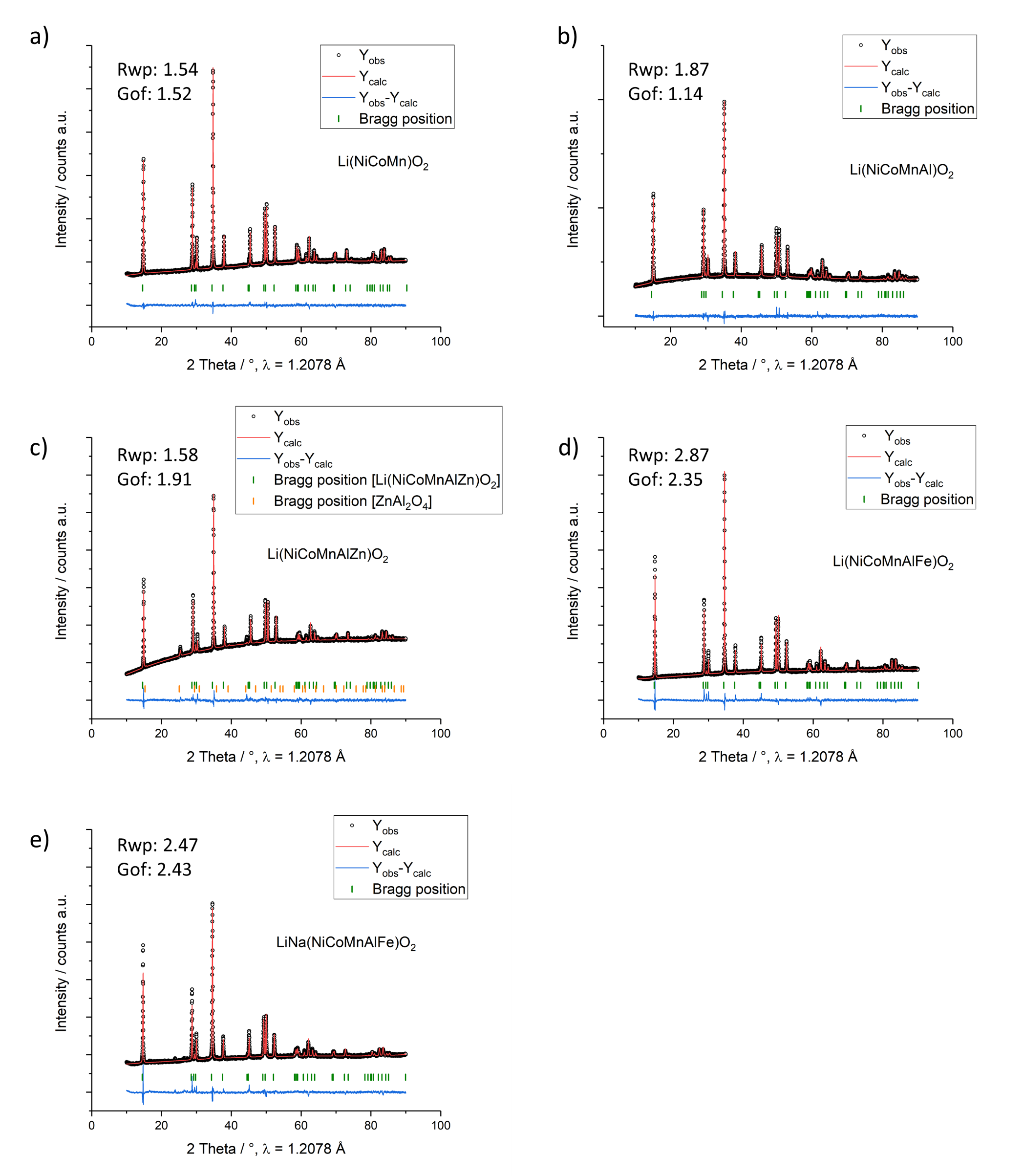


**Figure S1:** Rietveld analysis of XRD data for the different L-HEO compounds.

**Rietveld refinement analysis:**

The position of (transition) metals in the initial NCM structure was probed by carrying out refinements using different structural models (regarding the occupation of the 3*a* (Li) and 3*b* (M) sites by Li and M). Meaningful results were only obtained when assuming intermixing with Ni at the Li position. Hence, **Table 1** only presents the Li/Ni mixing. (Data obtained using Topas Academis V5 Software; Copyright 1992-2012 Alan A. Coelho, http://www.topas-academic.net/)


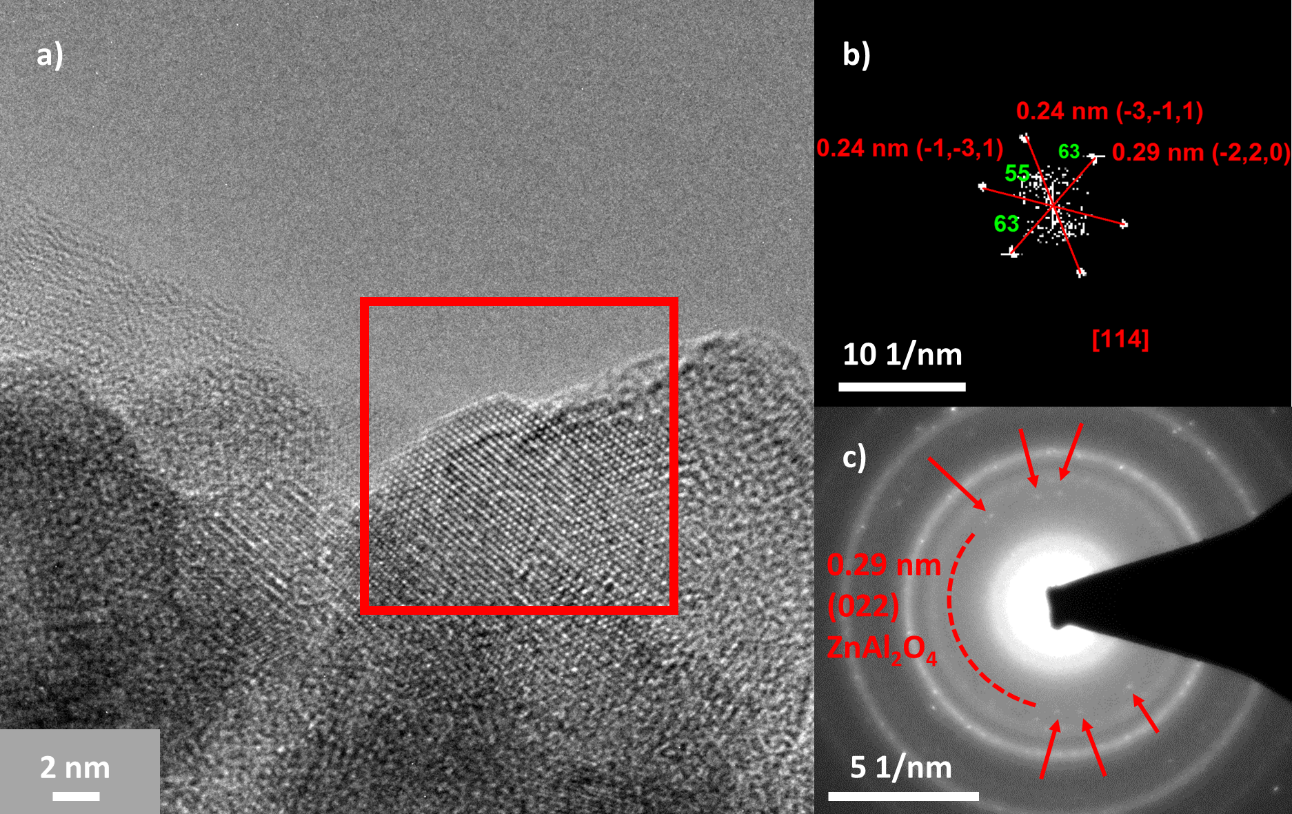


**Figure S2:** a) HR-TEM, b) FFT and c) SAED measurements indicate the presence of a distorted ZnAl_2_O_4_-type spinel structure.


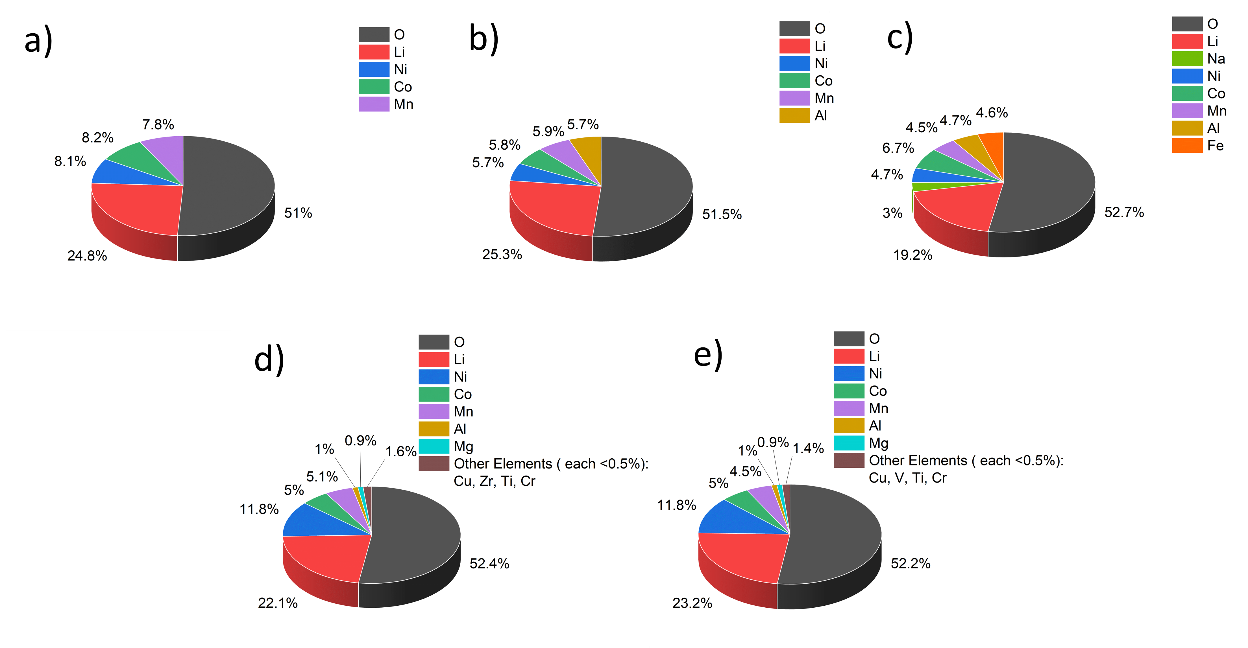


**Figure S3:** ICP-OES results for a) Li(NiCoMn)_1_O_2_, b) Li(NiCoMnAl)_1_O_2_, c) Li(NiCoMnAlFe)_1_O_2_, d) Li(Ni_0.46_Co_0.2_Mn_0.2_Mg_0.04_Al_0.04_Cr_0.015_Ti_0.015_Zr_0.015_Cu_0.015_)_1_O_2_ and e) Li(Ni_0.47_Co_0.2_Mn_0.18_Mg_0.04_Al_0.04_Cr_0.02_Ti_0.02_V_0.015_Cu_0.015_)_1_O_2_. The values are given in at%.


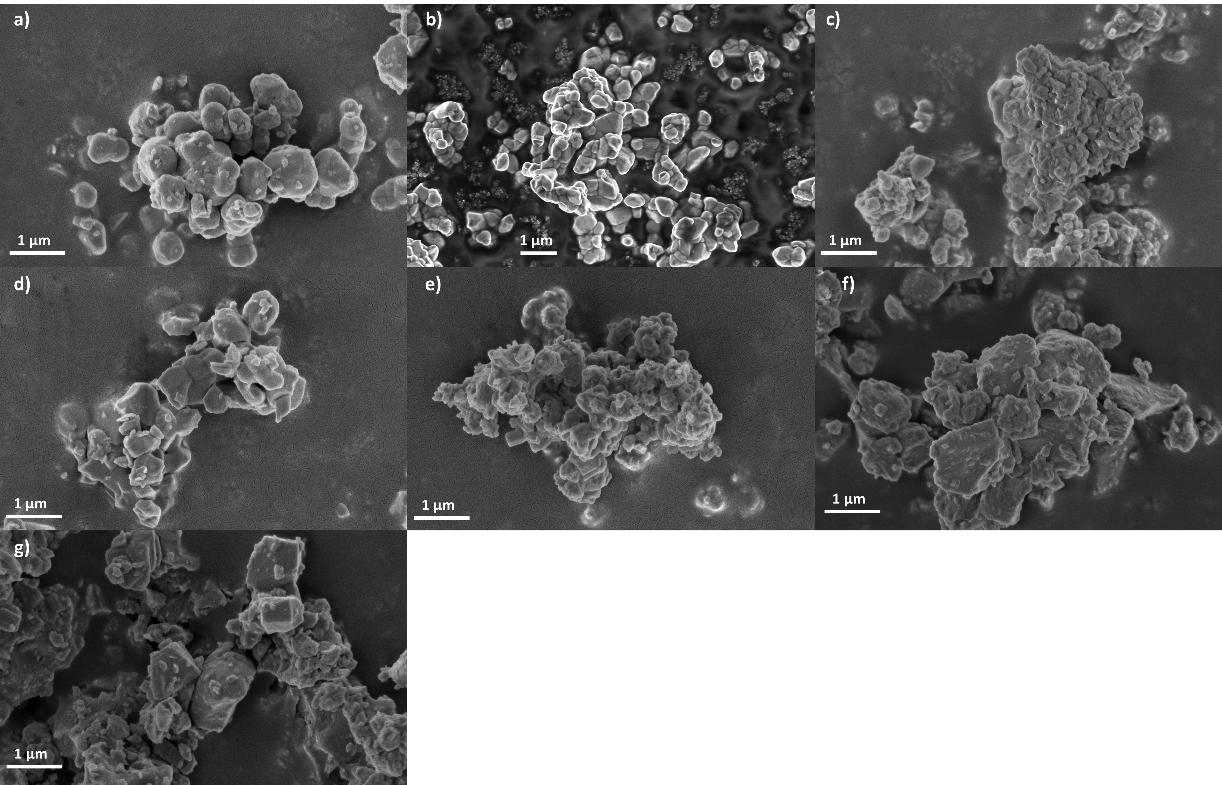


**Figure S4:** SEM micrographs of a) Li(NiCoMn)_1_O_2_, b) Li(NiCoMnAl)_1_O_2_, c) Li(NiCoMnAlZn)_1_O_2_, d) Li(NiCoMnAlFe)_1_O_2_, e) LiNa(NiCoMnAlFe)_1_O_2_,
f) Li(Ni_0.46_Co_0.2_Mn_0.2_Mg_0.04_Al_0.04_Cr_0.015_Ti_0.015_Zr_0.015_Cu_0.015_)_1_O_2_ and g) Li(Ni_0.47_Co_0.2_Mn_0.18_Mg_0.04_Al_0.04_Cr_0.02_Ti_0.02_V_0.015_Cu_0.015_)_1_O_2_.

**Entropy calculation:**

The calculation of configurational entropy for high-entropy ceramics is controversially discussed in literature. We used the common way, as reported in the original article about entropy-stabilized materials from Rost *et al*. and others. Here, the cation and anion sublattices are treated separately. The configurational entropy is derived from the Boltzmann entropy and can be determined according to:

$$S_{\mathrm{config}}= -R\sum_{i=1}^{N} x_{i}\ln x_{i}$$

Using this formula, the configurational entropy of alloys can be calculated. If the structure contains more than one sublattice, they need to be treated separately according to:

$$S_{\mathrm{config}}= -R[({\sum_{i=1}^{N} x_{i}\ln x_{i})}_{cation-site}+ ({\sum_{j=1}^{N} x_{j}\ln x_{j})}_{anion-site}]$$

Because the high-entropy oxides only contain oxygen (anion sublattice), the contribution from the second term equals 0. In this particular case, the calculation is as follows:

$$S_{\mathrm{config}}= -R[({\sum_{i=1}^{N} x_{i}\ln x_{i})}_{cation-site}+ ({\sum_{j=1}^{N} x_{j}\ln x_{j})}_{anion-site}]$$

$$S_{\mathrm{config}}= -R[({\sum_{i=1}^{N} x_{i}\ln x_{i})}_{cation-site}+ 0]$$

$$S_{\mathrm{config}}= -R[({\sum_{i=1}^{N} x_{i}\ln x_{i})}_{cation-site}]$$

For Li_0.8_Na_0.2_(NiCoMnAlFe)_1_O_2_ (Li_0.8_Na_0.2_Ni_0.2_Co_0.2_Mn_0.2_Al_0.2_Fe_0.2_O_2_) as an example, the formula needs to be normalized to 1 since every element sits on the same sublattice. Hence, it can be written as: *S*_config_ = −*R*[6·(0.1·ln0.1) + (0.4·ln0.4)] = 1.75R

**Table S1:** Calculated configurational entropies.

| Compound | Configurational entropy |
| --- | --- |
| Li(NiCoMn)_1_O_2_ | 1.10*R* |
| Li(NiCoMnAl)_1_O_2_ | 1.39*R* |
| Li(NiCoMnAlZn)_1_O_2_ | 1.61*R* |
| Li(NiCoMnAlFe)_1_O_2_ | 1.61*R* |
| LiNa(NiCoMnAlFe)_1_O_2_ | 1.75*R* |
| Li(Ni_0.47_Co_0.2_Mn_0.18_Mg_0.04_Al_0.04_Cr_0.02_Ti_0.02_V_0.015_Cu_0.015_)O_2_ | 1.53*R* |
| Li(Ni_0.46_Co_0.2_Mn_0.2_Mg_0.04_Al_0.04_Cr_0.015_Ti_0.015_Zr_0.015_Cu_0.015_)O_2_ | 1.51*R* |


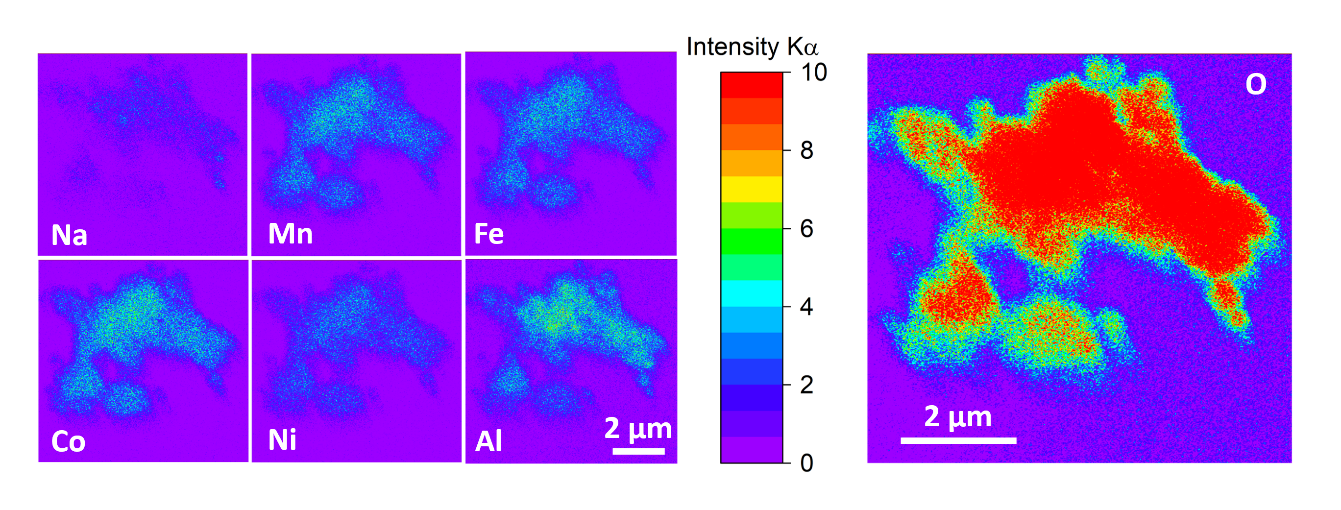


**Figure S5:** Elemental heat maps of a µm-sized LiNa(NiCoMnAlFe)_1_O_2_ agglomerate. The intensity of the Kα signals is shown on the scale between 1 and 10 au. The maps demonstrate that the elements are uniformly distributed. Areas of lower intensity result from lower sample thicknesses. The relatively lower intensity of the Na map is due to its low elemental weight and the respective interactions during EDX analysis (vice versa for O). The quantitative results indicate equimolar distribution of the transition metals, Al and Na.


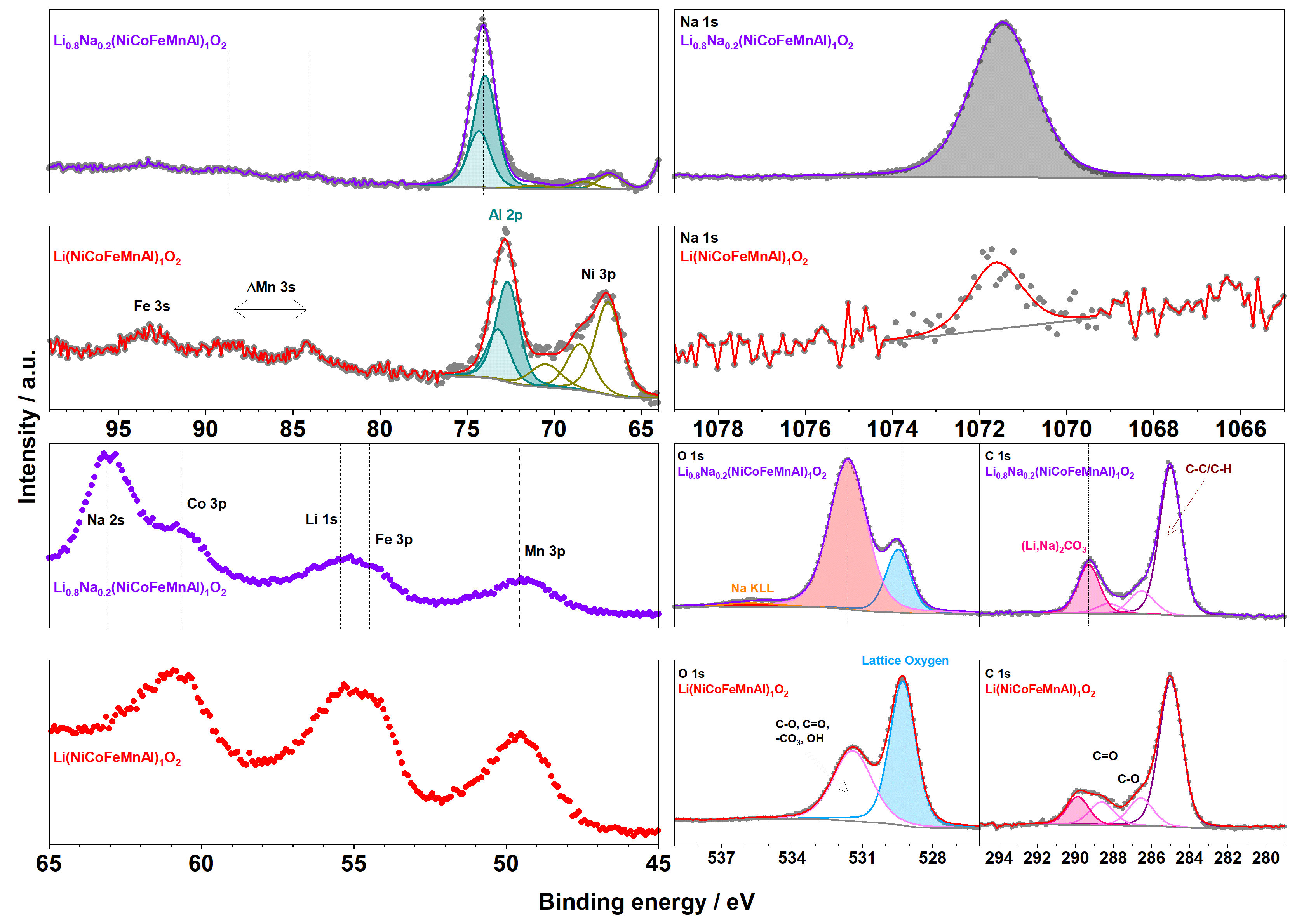

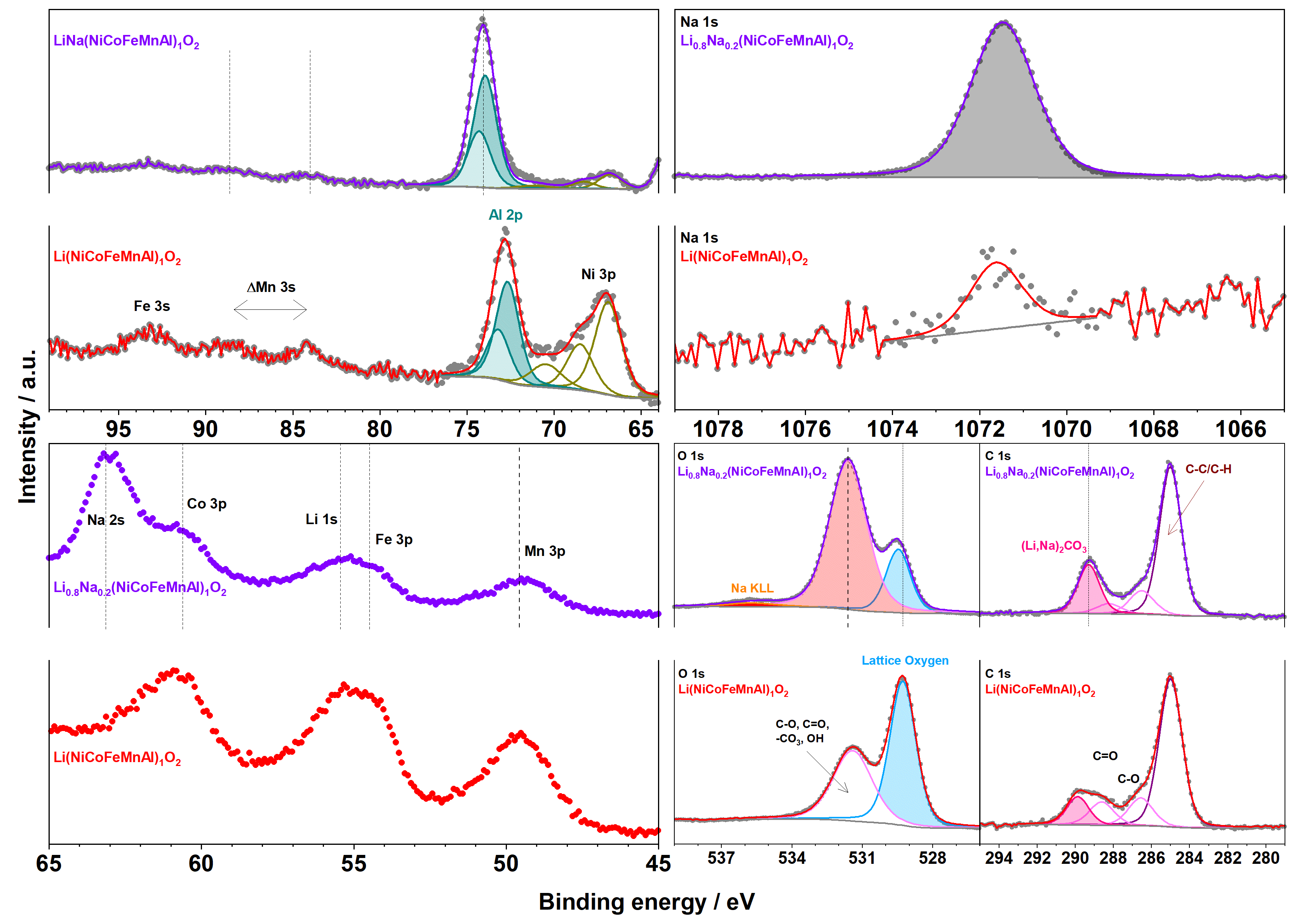


**Figure S6:** Al 2p detailed spectra of L-HEOs. Our attempt to properly deconvolute the Al 2p spectra (due to partial overlapping with the Ni 3p spectra) resulted in the peak fitting in cyan. The binding energy of Al 2p_3/2_ is 72.8 and 74 eV for Li(NiCoFeMnAl)_1_O_2_ and LiNa(NiCoFeMnAl)_1_O_2_, respectively.


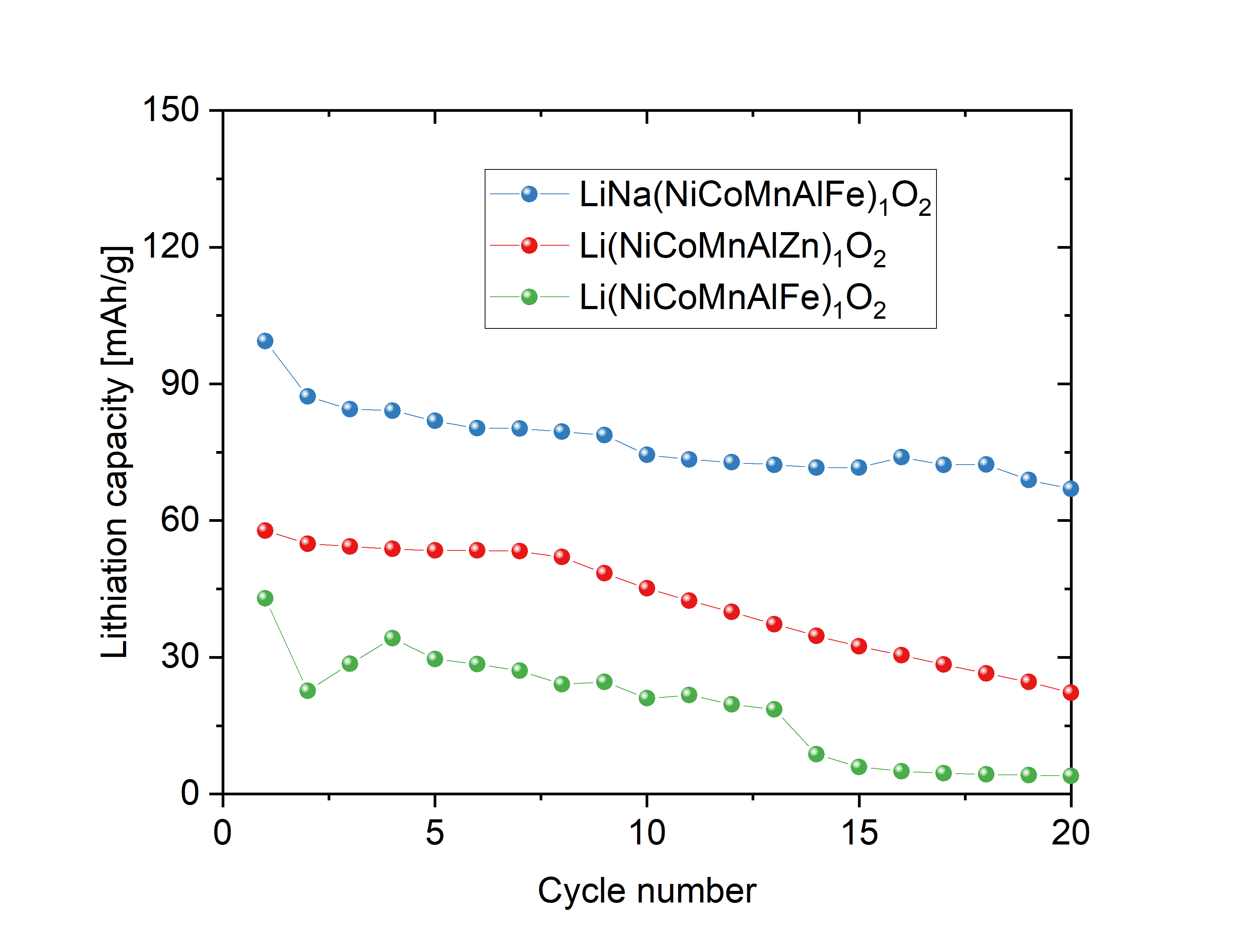


**Figure S7:** Specific discharge capacity from galvanostatic cycling experiments. The performance of LiNa(NiCoMnAlFe)_1_O_2_, Li(NiCoMnAlZn)_1_O_2_ and Li(NiCoMnAlFe)_1_O_2_ was tested in the voltage range of 3-4.5 V vs Li^+^/Li at a current rate of 0.1C (20 mA/g).


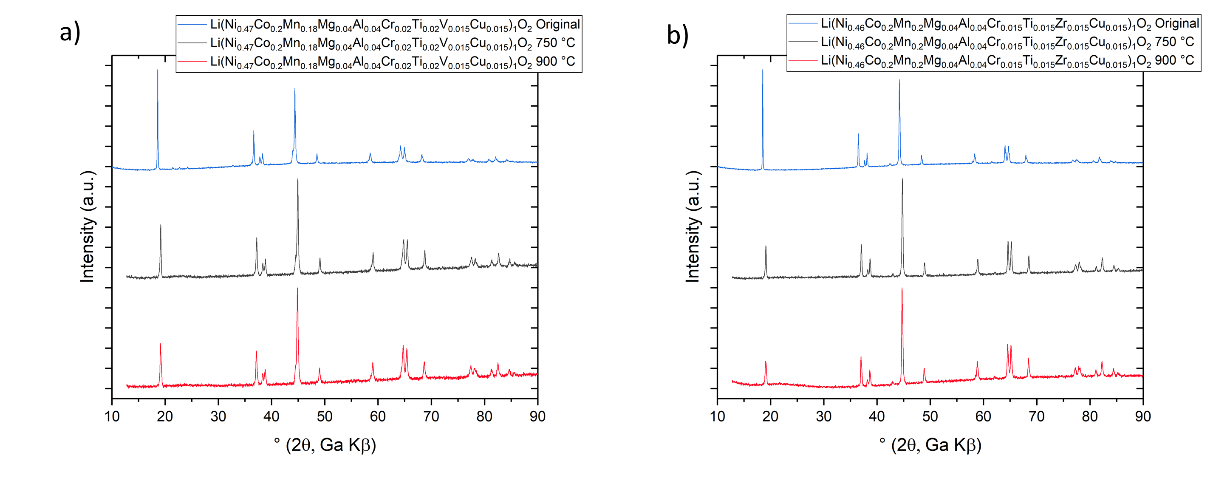


**Figure S8:** Results from heating tests conducted on a) Li(Ni_0.47_Co_0.2_Mn_0.18_Mg_0.04_Al_0.04_Cr_0.02_Ti_0.02_V_0.015_Cu_0.015_)O_2_ and b) Li(Ni_0.46_Co_0.2_Mn_0.2_Mg_0.04_Al_0.04_Cr_0.015_Ti_0.015_Zr_0.015_Cu_0.015_)O_2_. No additional reflections appeared upon heating, but the 003 to 104 intensity ratio changed significantly, thus indicating strong cation mixing.

**Additional information on the synthesis:**

The precursor solution was continuously delivered into the nebulizer, located inside a glass chamber at a rate of about 120 ml/h. The mist containing fine droplets was transported by flowing nitrogen, controlled by a MKS mass flow controller, into the hot zone of a tubular furnace. The internal diameter of the tube furnace was 1.8 cm and the heating length was around 40 cm, where nanoparticles were formed at a temperature of 800 °C. The pressure during synthesis was around 900 mbar. Finally, the collected powder was annealed at 900 °C for 12 h. The NSP setup used is shown in **Figure S9**. More details are provided elsewhere (see R. Djenadic, M. Botros, C. Benel, O. Clemens, S. Indris, A. Choudhary, T. Bergfeldt and H. Hahn, *Solid State Ionics*, **2014**, *263*, 49-56).

*
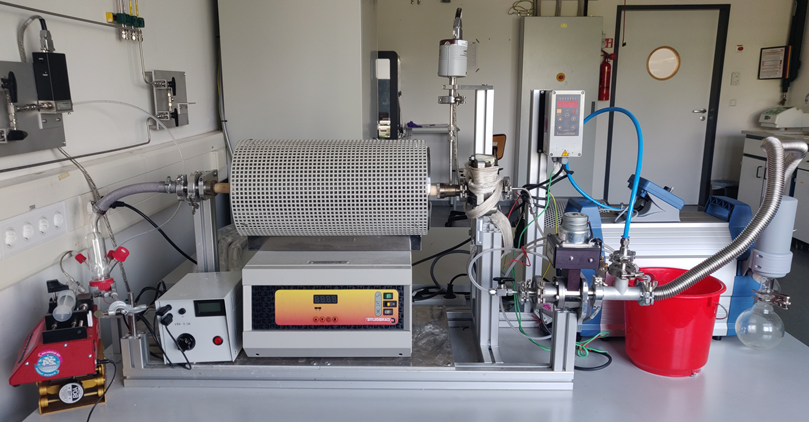
*

**Figure S9:** Photograph of the NSP setup used.
